# Clinical Trials Examining Deep Brain Stimulation for the Treatment of Epilepsy: An Analysis of the Current State

**DOI:** 10.7759/cureus.97571

**Published:** 2025-11-23

**Authors:** Jacob Gould, Saarang Patel, Bipin Chaurasia

**Affiliations:** 1 Krieger School of Arts and Sciences, Johns Hopkins University, Baltimore, USA; 2 Neurosurgery, Seton Hall University, South Orange, USA; 3 Neurosurgery, College of Medical Sciences, Bharatpur, NPL

**Keywords:** clinical trials, clinicaltrials.gov, deep brain stimulation, epilepsy, neurosurgery

## Abstract

Deep brain stimulation (DBS) has emerged as a consideration for patients with drug-resistant epilepsy who are not candidates for surgical resection. However, the landscape of DBS research in epilepsy remains fragmented across targets and study designs. This study aimed to systematically characterize all human DBS trials for epilepsy registered on ClinicalTrials.gov over the past two decades. A cross-sectional registry-based search of ClinicalTrials.gov was conducted in December 2023 using the terms “Epilepsy,” “Epilepsies,” or “Epileptic” combined with “Deep brain stimulation.” Eligible studies involved human participants and evaluated DBS as a therapeutic or diagnostic intervention. Extracted data included country, study type, enrollment, stimulation target, phase, and recruitment status. Descriptive statistics summarized study characteristics and temporal trends. Thirty-one trials met the inclusion criteria from 2003 to 2023. Most studies were interventional (80.6%), small to moderate in size (median enrollment 16), and predominantly conducted in the United States (51.6%). The thalamus was the most common target (45%), especially the anterior nucleus, followed by hippocampal and limbic sites. DBS of the anterior nucleus of the thalamus is currently the only FDA-approved DBS target for epilepsy, whereas all other stimulation sites remain investigational or off-label. Trial initiation increased steadily after 2015, peaking in 2021-2022. Only 25% of studies reported a formal phase designation, and few included pediatric populations. DBS research for epilepsy is expanding globally, with the number of registered DBS trials for epilepsy increasing more than threefold from 2003 to 2023, but research remains limited by small sample sizes and heterogeneous protocols. Thalamic stimulation continues to dominate current investigation, yet emerging targets reflect growing interest in network-level modulation. Standardized, multicenter trials are needed to define optimal targets and long-term efficacy in diverse patient populations.

## Introduction and background

Epilepsy is a common and heterogeneous neurological disorder characterized by recurrent, unprovoked seizures. Despite the advent of multiple new antiseizure medications over recent decades, approximately 30-35 % of patients with epilepsy develop drug-resistant epilepsy, which is defined as failure to achieve sustained seizure freedom with appropriate trials of two or more antiseizure medications in tolerated combinations [1]. Persistent seizures in drug-resistant epilepsy are associated with impaired quality of life, elevated risk of injury, cognitive decline, and increased mortality (including sudden death) [1]. In cases where the epileptogenic focus is localizable, surgical resection may provide benefit; however, many patients are not ideal surgical candidates due to multifocal onset and eloquent cortex involvement [2]. Surgical resection remains the gold standard for patients with a well-localized epileptogenic focus, yet only an estimated 20-30% of those with drug-resistant epilepsy are ultimately deemed eligible for curative resection following comprehensive presurgical evaluation. Among the remainder, a substantial proportion, up to 70-80%, have multifocal or poorly localized seizure onset zones or foci within eloquent cortex that preclude safe removal.

In light of these limitations, neuromodulation approaches have emerged as promising alternatives. Among them, deep brain stimulation (DBS) offers a reversible and programmable means to modulate the neural circuitry implicated in seizure initiation and propagation. Originally, DBS was developed and validated for movement disorders like Parkinson’s disease, but DBS has since been applied to a broader range of neurologic and psychiatric conditions (i.e., obsessive-compulsive disorder and depression) and is now being studied more intensively in epilepsy [3]. In the epilepsy domain, DBS is often considered in patients who are not candidates for resective surgery or when seizure foci are distributed or suboptimal for removal [4]. Patients may be considered poor candidates for resective surgery for several reasons, including seizure onset zones that are bilateral, multifocal, or overlap eloquent cortex, or when seizure localization remains uncertain despite comprehensive presurgical evaluation.

In epilepsy, the thalamus and limbic structures such as the hippocampus and amygdala have been the most frequently studied targets for DBS [5]. The Stimulation of the Anterior Nucleus of the Thalamus for Epilepsy (SANTE) trial and subsequent studies have demonstrated meaningful seizure reductions in selected patients [6]. Notably, based on evidence from the SANTE trial, DBS of the anterior nucleus of the thalamus (ANT) received U.S. Food and Drug Administration (FDA) approval in 2018 for use in adults with drug-resistant focal epilepsy. In contrast, other DBS targets such as the hippocampus, amygdala, and fornix remain investigational and are currently explored in off-label or research settings. However, the overall evidence base remains limited by small sample sizes, heterogeneous methodologies, and few randomized trials. To better understand the evolution of this field, we conducted a systematic analysis of all human DBS trials for epilepsy from 2003 to 2023 registered on ClinicalTrials.gov and characterized them by their geographic distribution, design features, institutional representation, and stimulation targets to bring clarity to the current state and identify gaps in ongoing research.

## Review

Methods

This study was designed as a cross-sectional, registry-based analysis of publicly available clinical trial data. A comprehensive search of the ClinicalTrials.gov database was performed in December 2023 to identify all clinical studies investigating DBS for the treatment of epilepsy. The search was conducted using the following conditions and disease terms: “Epilepsy,” “Epilepsies,” and “Epileptic” in combination with the intervention and treatment term “Deep brain stimulation.” No date or status restrictions were applied, and all results were included from database inception to the date of search. Studies were included if they: (1) involved human participants diagnosed with epilepsy; (2) evaluated DBS as a therapeutic or diagnostic intervention; and (3) were listed as interventional or observational studies. Studies were excluded if they: (1) were preclinical or animal-based; (2) investigated DBS for other neurological or psychiatric conditions without an epilepsy cohort or investigated non-DBS epileptic therapies; or (3) were duplicate entries or lacked sufficient study details. When duplicate or overlapping trial entries were identified, only the most complete and up-to-date record was retained. Trials with ambiguous or incomplete data were noted but excluded from quantitative analyses.

For each eligible study, the following data was extracted: ClinicalTrials.gov identifier (NCT number), study title, country of origin, study start year, study type (interventional or observational), funding source, trial phase, recruitment status, enrollment size, stimulation target, type of epilepsy studied, population age range, and availability of published results. Data extraction was performed manually from trial registry entries to ensure accuracy and consistency. Data compilation and analysis were performed in Microsoft Excel. As ClinicalTrials.gov data is self-reported, the extracted information was limited to the completeness of what was reported.

**Figure 1:**
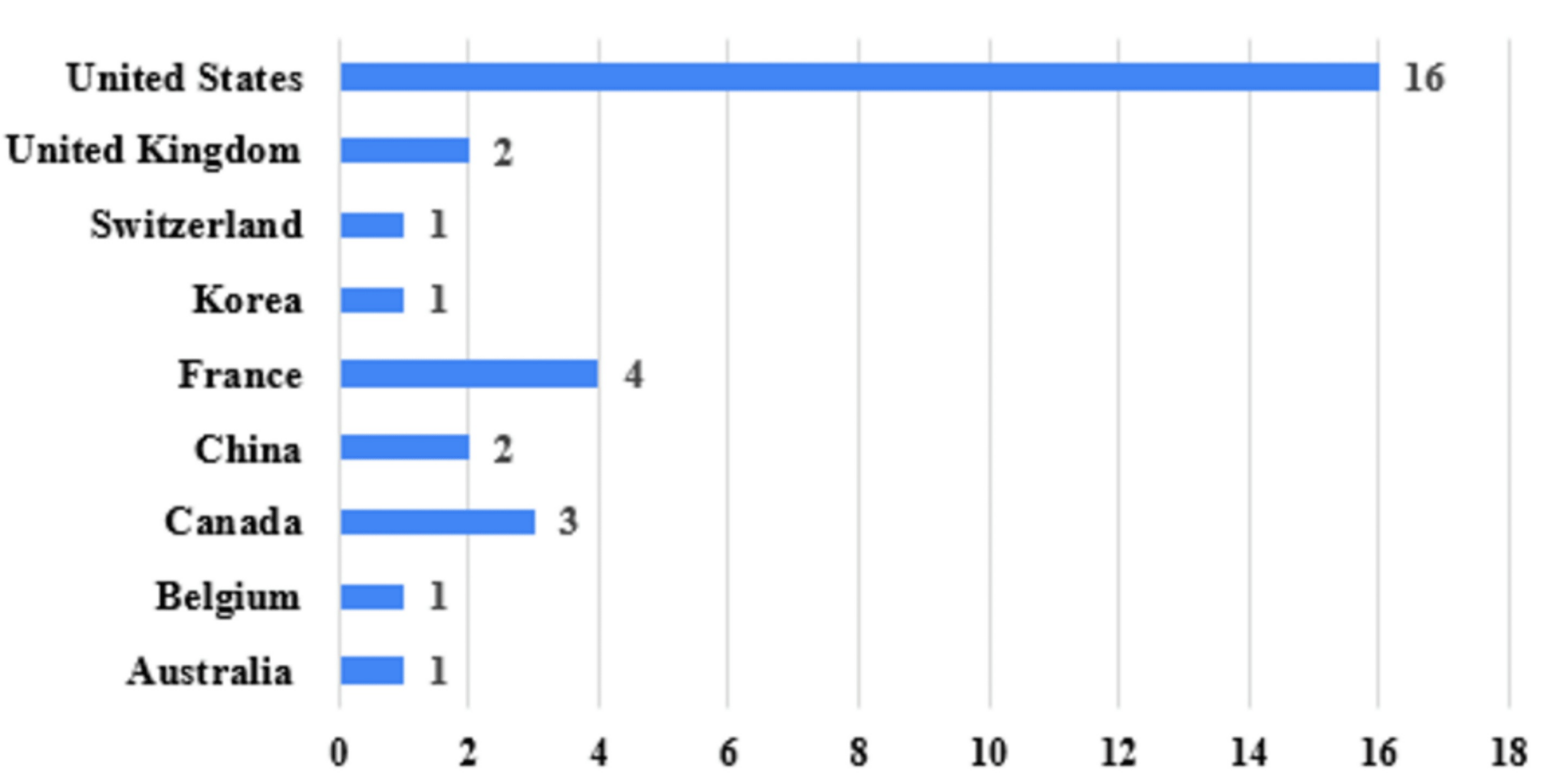
Clinical Trials by Country Clinical trials organized by country of origin.

**Figure 2:**
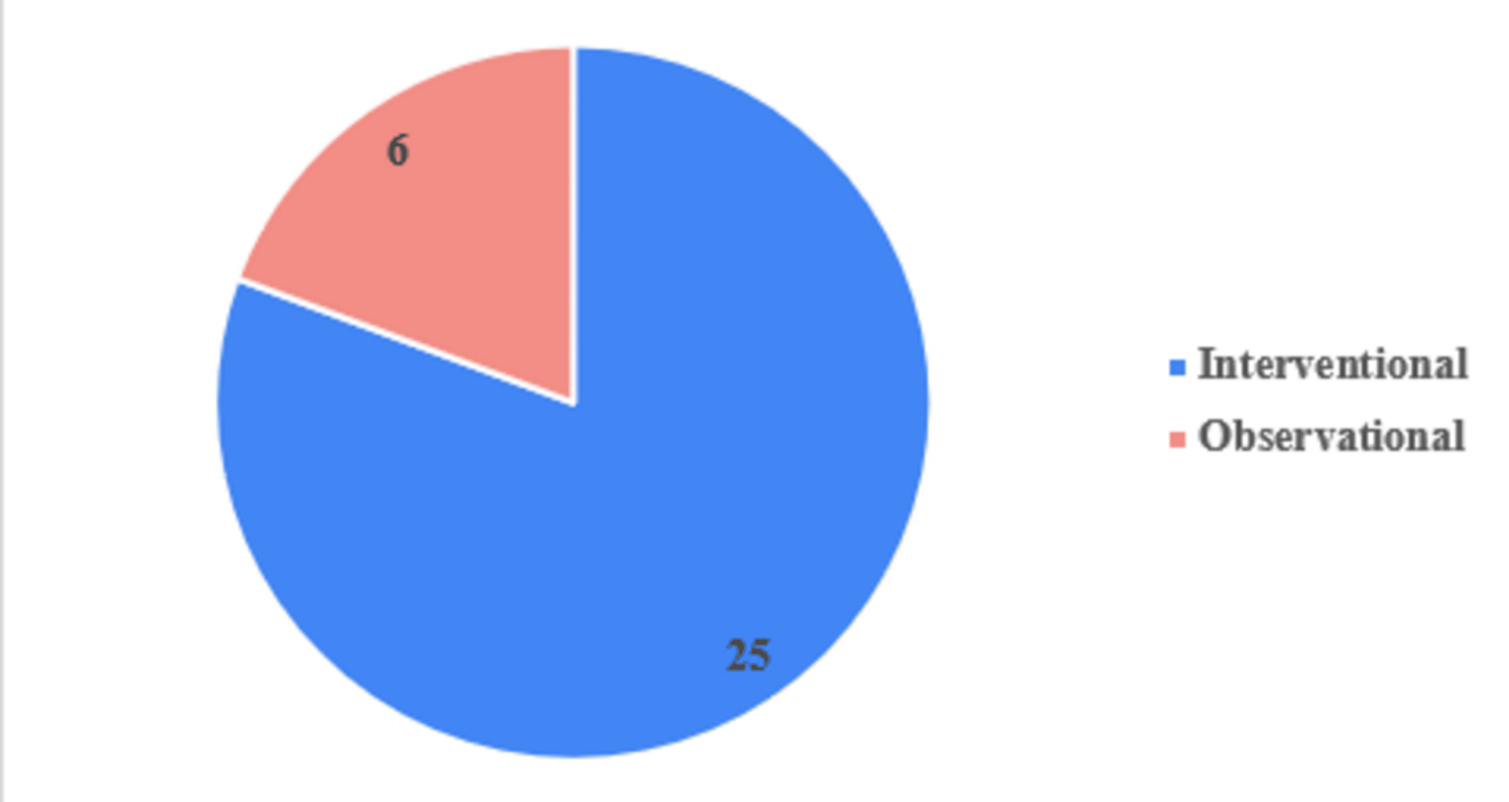
Clinical Trials Compared by Study Type Clinical trials compared by study type.

**Figure 3:**
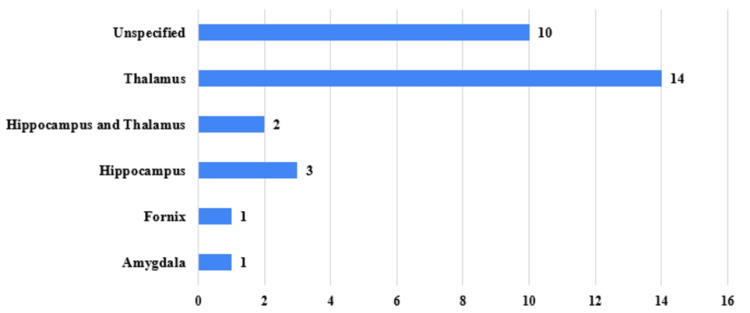
Key Stimulated Regions Across Clinical Trials Key stimulated regions across included clinical trials.

Descriptive statistics were used to summarize study characteristics. Continuous variables such as enrollment size were reported as means or medians when applicable, and categorical variables were summarized as frequencies and percentages. Trends were evaluated across geographic distribution, study type, stimulation target, and temporal patterns in study initiation. Figures were generated to illustrate: (1) the distribution of trials by country (Figure 1), (2) the proportion of interventional versus observational studies (Figure 2), and (3) the distribution of stimulation targets across studies (Figure 3).

Results

A total of 43 trials were retrieved from ClinicalTrials.gov, of which 31 met inclusion criteria after exclusion of preclinical, non-human, non-DBS, or duplicate studies (Table 1) [7-37]. Included trials were initiated between 2003 and 2023, reflecting two decades of DBS investigation in epilepsy. The majority of studies (16/31, 51.6%) were conducted in the United States with the remainder distributed across Europe, Asia, and South America. The United Kingdom, China, and France each accounted for multiple registered studies, whereas other nations such as Australia, Belgium, and Korea contributed single trials (Figure 1). Several North American academic centers and private medical device companies appeared repeatedly across the dataset, including the Mayo Clinic, the Hospital of Sick Children (Canada), and MedtronicNeuro.

**Table 1:** Summary of Deep Brain Stimulation Clinical Trials Table providing a summary of the included deep brain stimulation clinical trials.

ClinicalTrials.gov ID	Study Title	Country of Origin	Institution	Start Year	Enrollment Size	Study Type	Study Description	Population Age	Type of Epilepsy	Brain Region Stimulated
NCT05437393 [7]	Children's Adaptive Deep Brain Stimulation for Epilepsy Trial (CADET): Pilot	United Kingdom	University College, London	2023	4	Interventional	Deep Brain stimulation using a novel device: Bioinduction "Picostim" Deep Brain Stimulation system	5 years to 14 years (Child)	Lennox-Gastaut Syndrome	Centromedian nucleus (bilateral)
NCT04164056 [8]	Hippocampal and Thalamic DBS for Bilateral Temporal Lobe Epilepsy	China	Zhejiang University	2019	80	Interventional	Deep brain stimulation on the hippocampus or the anterior nucleus of the thalamus	12 years to 60 years (Child, Adult)	Bilateral temporal lobe epilepsy	Hippocampal and thalamic
NCT04692701 [9]	Pulvinar Stimulation in Epilepsy: a Pilot Study	France	Hopitaux de marseille	2021	12	Interventional	Deep brain stimulation of the medial pulvinar	18 years to 60 years (Adult)	Focal or multifocal drug-resistant epilepsy	Medial pulvinar
NCT03465163 [10]	A Deep Brain Stimulation System in Epilepsy: Tracking Neural Excitability	Australia	St Vincent's Hospital Melbourne	2018	1	Interventional	The device is called the Medtronic Activa PC+S system. Two devices will be implanted per participant. The electrodes will be surgically implanted bilaterally into the hippocampus and anterior nucleus of the thalamus.	18 years and older (Adult)	Epilepsy with non-resectable pathologies	Hippocampus and anterior nucleus of the thalamus
NCT04181229 [11]	Deep Brain Stimulation Post Failed Vagal Nerve Stimulation (DBSpostVNS)	Canada	The Hospital of Sick Children	2019	50	Interventional	Patients will receive surgical implantation of the Medtronic DBS device (Device # 37601). Two (2) electrodes will be implanted bilaterally in the centromedian nucleus.	8 years to 18 years (Child, Adult)	Unspecified	Centromedian nucleus
NCT05011773 [12]	Manipulating and Optimising Brain Rhythms for Enhancement of Sleep (MORPHEUS)	United Kingdom	University of Oxford	2021	4	Interventional	Electrical pulses from an implanted generator that has already been implanted for therapeutic reasons	18 years to 85 years (Adult)	Unspecified	Unspecified
NCT02383407 [13]	Low Frequency Electrical Stimulation of the Fornix in Intractable Mesial Temporal Lobe Epilepsy (MTLE) (MTLE-DBS)	United States	George Washington University	2013	6	Interventional	Low-frequency electrical stimulation of the fornix (LFSF) in participants with medically intractable mesial temporal lobe epilepsy. Secondary aims include evaluation of psychiatric changes, seizure frequency, and quality of life during LFSF.	18 years to 65 years (Adult)	Intractable mesial temporal lobe epilepsy	Fornix
NCT01210781 [14]	Target Planning for Placement of DBS-electrodes and Follow-up of the Clinical Efficacy of Stimulation	Switzerland	University of Zurich	2009	500	Observational	Describe the variability of target coordinates in the patient group and to relate it to clinical outcome as documented in standardized questionnaires.	18 years to 90 years (Adult)	Unspecified	Unspecified
NCT01590862 [15]	ON/​OFF Stimulation and Reward Motivation in Patients With Deep Brain Stimulators	United States	Massachusetts General Hospital	2021	60	Interventional	Assess reward motivation behavior with Medtronic deep brain stimulation on and off	18 years to 70 years (Adult)	Unspecified	Unspecified
NCT05418894 [16]	Mapping and Modulating the Spatiotemporal Dynamics of Socio-Affective Processing	United States	Baylor College of Medicine	2022	84	Interventional	First-in-human intracranial neural recording opportunities created by a novel therapeutic platform termed "stereotactic electroencephalography-informed deep brain stimulation" (stereo-EEG-informed DBS), as well as the powerful platform of intracranial stereotactic recording and stimulation in patients undergoing epilepsy surgical evaluation	22 years to 70 years (Adult)	Unspecified	Unspecified
NCT02602899 [17]	Deep Brain Stimulation of the Anterior Nucleus of the Thalamus in Refractory Epilepsy (ANT-DBS-RE)	China	Beijing Tiantan Hospital	2015	30	Interventional	Evaluate the long-term clinical effectiveness and safety of the PINS Deep Brain Stimulation in patients with refractory epilepsy.	12 years to 60 years (Child, Adult)	Refractory epilepsy	Anterior nucleus of the thalamus
NCT02076698 [18]	Deep Brain Stimulation of the Anterior Nucleus of the Thalamus in Epilepsy	France	University Hospital, Grenoble	2014	62	Interventional	Assess the clinical efficacy of DBS on epilepsy according to its number and severity at 1-year follow-up.	16 years to 60 years (Child, Adult)	Pharmacoresistant partial epilepsy	Anterior nucleus of the thalamus
NCT03870308 [19]	fMRI of Active DBS Stimulation in Epilepsy	United States	Mayo Clinic	2019	6	Interventional	Functional MRI (fMRI) scanning to reveal a pattern in brain activity during Deep Brain Stimulation (DBS), which will correlate to possible seizure freedom.	18 years and older (Adult)	Refractory epilepsy	Anterior thalamic nucleus
NCT03900468 [20]	Medtronic Deep Brain Stimulation (DBS) Therapy for Epilepsy Post-Approval Study (EPAS) (EPAS)	United States	MedtronicNeuro	2020	140	Interventional	Evaluate the long-term safety and effectiveness of Medtronic DBS therapy for epilepsy on seizure reduction in newly implanted participants through 3 years of follow-up.	18 years and older (Adult)	Refractory epilepsy	Unspecified
NCT05600738 [21]	Network Effects of Therapeutic Deep Brain Stimulation	United States	The University of Texas Health Science Center, Houston	2022	25	Interventional	Map the acute, short-term cortical evoked responses to thalamic electrical stimulation in persons with intractable epilepsy	18 years and older (Adult)	Intractable epilepsy	Thalamus
NCT05292183 [22]	Modulation of Emotion Perception in Humans Via Amygdala Stimulation	United States	Dartmouth-Hitchcock Medical Center	2022	16	Interventional	Patients with epilepsy who are being evaluated for epilepsy surgery and have intracranial EEG electrodes. In this study, the aim is to record brain signals from areas important in social and emotional processing and to understand how electrical brain stimulation - called neuromodulation - affects such processing.	18 years and older (Adult)	Unspecified	Amygdala
NCT00194870 [23]	Electroencephalography (EEG) and Deep Brain Stimulation (DBS) in Epilepsy	United States	Weill Medical College of Cornell University	2003	5	Interventional	To assess EEG changes during electrical stimulation of the thalamus to treat people with epilepsy	18 years to 65 years (Adult)	Intractable epilepsy	Anterior nucleus of the thalamus
NCT04753983 [24]	A Study to Evaluate fMRI of Active DBS Stimulation in Epilepsy	United States	Mayo Clinic	2022	0	Interventional	Functional imaging to study the mechanisms of the anterior nucleus of the thalamus (ANT) deep brain stimulation (DBS).	18 years and older (Adult)	Refractory epilepsy	Anterior thalamic nucleus
NCT05493722 [25]	Optimization of Deep Brain Stimulation Parameters in Patients With Medically Refractory Epilepsy	United States	University of Minnesota	2023	20	Interventional	This study will develop a platform for stimulation setting optimization based on power spectral density (PSD) measures.	18 years and older (Adult)	Refractory epilepsy	Unspecified
NCT04286776 [26]	Using Direct Brain Stimulation to Study Cognitive Electrophysiology	United States	University of Pennsylvania	2019	250	Interventional	Employing direct electrical brain stimulation as a tool to study those dynamics and their relationship to memory performance.	18 years to 65 years (Adult)	Refractory epilepsy	Unspecified
NCT00717431 [27]	A Multicenter Study of Hippocampal Electrical Stimulation (HS) in Mesial Temporal Lobe Epilepsy (METTLE)	Canada	University of Calgary	2008	8	Interventional	To determine whether hippocampal electrical stimulation (HS) is safe and more effective than simply implanting an electrode in the hippocampus without electrical stimulation (HI), in patients with mesial temporal lobe epilepsy	18 years and older (Adult)	Unilateral or bilateral mesial temporal lobe epilepsy.	Hippocampal
NCT01521754 [28]	Product Surveillance Registry- Deep Brain Stimulation for Epilepsy (MORE)	United States	MedtronicNeuro	2012	191	Observational	Evaluate the long-term effectiveness, safety and performance of market-released Medtronic Neuromodulation products for deep brain stimulation (DBS) for the treatment of refractory epilepsy	18 years and older (Adult)	Unspecified	Unspecified
NCT01141764 [29]	Cerebral Metabolic Changes Associated With Thalamic Stimulation	Canada	The Hospital of Sick Children	2010	4	Interventional	Evaluate the brain circuits' function and circuits involved in the mechanism of thalamic DBS in patients with medically refractory epilepsy.	18 years to 85 years (Adult)	Refractory epilepsy	Thalamus
NCT04771065 [30]	Deep Brain Stimulation of the Anterior Nucleus of the Thalamus in Intractable Epilepsy	France	University Hospital, Montpellier	2021	10	Observational	Microendoscopic transventricular deep brain stimulation of the anterior nucleus of the thalamus as a safe and efficient treatment in intractable epilepsy	18 years and older (Adult)	Refractory epilepsy	Anterior nucleus of the thalamus
NCT00101933 [31]	SANTE - Stimulation of the Anterior Nucleus of the Thalamus for Epilepsy	United States	MedtronicNeuro	2003	157	Interventional	Safety and effectiveness of bilateral stimulation of the anterior nucleus of the thalamus as adjunctive therapy for reducing the frequency of seizures in adults diagnosed with epilepsy characterized by partial-onset seizures, with or without secondary generalization, that are refractory to antiepileptic medications.	18 years to 65 years (Adult)	Refractory epilepsy	Anterior nucleus of the thalamus
NCT04897776 [32]	Stimulation of the Thalamus for Arousal Restoration in Temporal Lobe Epilepsy (START)	United States	Yale University	2021	5	Interventional	Thalamic stimulation to prevent impaired consciousness in epilepsy	18 Years to 75 Years (Adult)	Temporal lobe epilepsy	Thalamus
NCT03404128 [33]	Long-term Follow-up of Hippocampal DBS for Refractory Epilepsy	Belgium	University Hospital, Ghent	2016	6	Observational	Long-term evaluation of the effects of hippocampal DBS on seizure frequency and cognition	(Child, Adult)	Refractory epilepsy	Hippocampal
NCT02235792 [34]	High Frequency Oscillations in Neurologic Disease	United States	Mayo Clinic	2014	7	Interventional	Evaluate the high-frequency range deep brain oscillations (HFO) as pathologic markers in patients undergoing deep brain stimulation for epilepsy or Parkinson's disease.	18 years to 75 years (Adult)	Unilateral or bilateral mesial temporal lobe (hippocampal) epilepsy with complex partial, and/or secondarily generalized seizures	Hippocampal
NCT00575081 [35]	Physiological Brain Atlas Development (Brain Atlas)	United States	Vanderbilt University Medical Center	2006	5000	Observational	Development of a physiological brain atlas registry that will allow us to significantly improve the data collection and use of physiological data in a normalized brain volume.	6 years to 90 years (Child, Adult)	Unspecified	Unspecified
NCT00228371 [36]	STIMEP: Assessment of Subthalamic Nucleus Stimulation in Drug Resistant Epilepsy	France	University Hospital, Grenoble	2005	4	Interventional	Evaluate the effectiveness and the safety of deep brain stimulation in drug-resistant epilepsy.	18 years to 50 years (Adult)	Refractory epilepsy	Subthalamic nucleus
NCT05535556 [37]	Comparison of the Electric Plasma Surgical Tool "PlasmaBlade" for Replacement of the Deep Brain Stimulation (DBS) Devices With Conventional Surgery	Korea	Yonsei University	2022	50	Observational	Comparison of the electric plasma surgical tool "PlasmaBlade" for replacement of the deep brain stimulation (DBS) devices with conventional surgery	19 years and older (Adult)	Unspecified	Unspecified

Most trials were interventional (25/31, 80.6%), while six (19.4%) were observational (Figure 2). Only eight (25.8%) studies reported an applicable phase designation, largely Phase III, which is consistent with late-stage confirmatory testing. Among all trials, 12 (38.7%) were actively recruiting at the time of analysis, 10 (32.3%) had been completed, and the remainder were either not yet recruiting or had unknown status. The initiation of DBS trials for epilepsy increased steadily over time. Early exploratory efforts in the 2000s gave way to a marked rise after 2015. The peak years for study initiation were 2021 and 2022, each with five trials, indicating accelerating research interest in the past few years. Notably, the early 2000s saw a predominance of small, single-center pilot studies, whereas more recent years show multicenter collaborations and device-based industry involvement.

Enrollment varied widely across studies, ranging from single-patient case investigations to large multicenter efforts with over 200 participants. The average enrollment size was 219 participants (SD = 893) across all trials, although the median was substantially lower (16), reflecting several large outliers. Twenty-four studies targeted adult populations aged ≥ 18 years only, six studies included mixed cohorts of both children and adults, and one study included only children. Pediatric-focused DBS trials remain uncommon, reflecting safety and ethical considerations in this population.

DBS targets were heterogeneous (Figure 3). The thalamus was the predominant site, reported in 14 studies with the anterior thalamic nucleus representing 7 of those trials. Additional targets included the hippocampus (n = 3), hippocampus + thalamus (n = 2), fornix (n = 1), and amygdala (n = 1). Ten studies did not specify the stimulation target, often reflecting early-phase trials. The predominance of thalamic targeting mirrors findings from prior pivotal studies such as the SANTE trial and underscores ongoing interest in network-level modulation of seizure propagation pathways. Trials encompassed a range of epileptic syndromes. Temporal lobe epilepsy (TLE) was explicitly designated in five studies (16%). Other trials targeted focal epilepsies of diverse origins as well as generalized or multifocal refractory epilepsies.

Discussion

This analysis provides an updated overview of all registered clinical trials investigating DBS for epilepsy. Over the past two decades, there has been a steady expansion of DBS research activity, particularly in the last five years, which reflects growing interest in neuromodulation as a therapeutic option for drug-resistant epilepsy [4]. Between 2003 and 2023, the number of registered DBS trials for epilepsy increased more than threefold, with over 60% initiated after 2015 and the highest activity observed in 2021-2022. The majority of identified studies were interventional, small to moderate in size, and predominantly conducted in high-resource academic centers within the United States and Europe. The thalamus, and specifically the anterior nucleus, emerged as the most common stimulation target, consistent with prior evidence supporting its role in seizure network modulation [6]. However, emerging trials exploring hippocampal, amygdalal, and fornical stimulation highlight ongoing efforts to define optimal targets for various epilepsy subtypes [5]. 

Compared with the existing literature, the predominance of thalamic targeting reflects continuity with seminal work from the SANTE trial, which demonstrated a 56% median seizure reduction at two years and established the anterior thalamus as a viable DBS target for focal epilepsy [38]. Similarly, subsequent long-term follow-ups have confirmed durable efficacy and acceptable safety profiles, although complete seizure freedom remains rare. Our observation that new studies are also investigating hippocampal, amygdalar, and fornical stimulation parallels smaller clinical series such as those by Velasco et al., which reported promising reductions in seizure frequency in TLE but with greater procedural variability [39].

Unlike earlier meta-analyses or narrative reviews that aggregate clinical outcomes, this registry-level analysis captures the structural evolution of the field by showing a clear shift toward multicenter collaborations, device-industry partnerships, and increasing trial diversity across continents. In our analysis, over half of all trials were conducted outside the United States or involved international collaboration, and the proportion of multicenter studies has steadily increased since 2018. Notably, several recent trials listed industry sponsorship or device manufacturer involvement, reflecting growing commercial and translational interest in DBS for epilepsy. Together, these findings underscore a gradual shift from early single-center feasibility studies toward coordinated, large-scale investigations designed to establish reproducible outcomes and regulatory evidence. However, persistent challenges mirror those cited by Jobst and Cascino and Sprengers et al.: small sample sizes, inconsistent endpoints, and heterogeneous stimulation parameters that limit cross-study comparability [40,41]. Our findings further reinforce the underrepresentation of pediatric and generalized epilepsy cohorts.

Future work should focus on standardizing clinical trial methodology, expanding multicenter collaborations, and reporting consistent outcome measures, which can help to enable meaningful comparisons across studies. There remains a need for larger, randomized controlled trials with long-term follow-up to define durable efficacy and safety [41]. Furthermore, broader inclusion of underrepresented groups including pediatric epilepsy populations is essential to fully delineate the therapeutic potential of DBS in epilepsy care.

Limitations

This study is limited by its reliance on ClinicalTrials.gov data, which is self-reported by investigators and may contain incomplete or outdated information. A notable limitation of this registry-based analysis is that several trials did not specify a stimulation target in publicly available records. Although DBS is inherently target-dependent, some registry entries, particularly device registries, parameter-optimization studies, or exploratory mapping trials, listed “unspecified” under the target field. We reviewed each record and related publications to verify missing information, but when no clear anatomical site was reported, we retained the designation as “unspecified.” This likely reflects incomplete public reporting rather than the absence of a defined target within the actual study protocol. Such omissions underscore the need for standardized reporting of DBS targets, electrode models, and programming parameters in trial registries to enhance reproducibility and facilitate meta-analytic synthesis. Additionally, lack of standardized reporting for stimulation parameters and outcomes precludes direct comparison between studies. Despite these limitations, this analysis provides a comprehensive snapshot of the current state of DBS clinical research for epilepsy and highlights opportunities to refine its application through coordinated, high-quality clinical investigation.

## Conclusions

DBS for epilepsy represents an evolving and increasingly global research frontier. While most registered trials remain small and exploratory, the growing number of multicenter and industry-supported studies signals maturation of the field and a movement toward standardization. The predominance of thalamic targeting reflects its established role in seizure network modulation, yet the emergence of trials exploring hippocampal and limbic circuitry highlights an expanding effort to individualize DBS therapy to specific epilepsy subtypes. Moving forward, adequately powered randomized controlled trials with consistent outcome metrics and broader inclusion of pediatric and generalized epilepsy populations will be essential to refine therapeutic efficacy and safety. Strengthening data transparency and harmonization across registries can further enable meaningful meta-analysis and regulatory evaluation. With coordinated and high-quality clinical investigation, DBS has the potential to transition from an experimental intervention to a routine and precise tool within the comprehensive management of drug-resistant epilepsy.
